# Neuropeptide FF Promotes Neuronal Survival and Enhances Synaptic Protein Expression Following Ischemic Injury

**DOI:** 10.3390/ijms252111580

**Published:** 2024-10-28

**Authors:** In-Ae Choi, Ji Hee Yun, Jongmin Lee, Dong-Hee Choi

**Affiliations:** 1 Center for Neuroscience Research, Institute of Biomedical Science and Technology, Konkuk University, Seoul 05029, Republic of Korea; inaes2@bu.ac.kr (I.-A.C.); bsea78@empas.com (J.H.Y.); leej@kuh.ac.kr (J.L.); 2 Department of Occupational Therapy, Division of Health, Baekseok University, Cheonan-si 31065, Chung-cheongnam-do, Republic of Korea; 3 Department of Rehabilitation Medicine, Konkuk University School of Medicine, Konkuk University, Seoul 05029, Republic of Korea; 4 Department of Medical Science, Konkuk University School of Medicine, Konkuk University, Seoul 05029, Republic of Korea

**Keywords:** Neuropeptide FF, ischemic injury, stroke recovery, synaptic plasticity, BDNF, PKCε, SIRT1, PPARγ

## Abstract

This study explores the neuroprotective effects of neuropeptide FF (NPFF, FLFQPQRFamide) in the context of ischemic injury. Based on transcriptomic analysis in stroke models treated with 5-Aza-dC and task-specific training, we identified significant gene expression changes, particularly involving NPFF. To further explore NPFF’s role in promoting neuronal recovery, recombinant NPFF protein (rNPFF) was used in primary mixed cortical cultures subjected to oxygen-glucose deprivation and reoxygenation. Our results demonstrated that rNPFF significantly reduced lactate dehydrogenase release, indicating decreased cellular damage. It also significantly increased the expression of TUJ1 and MAP2, markers of neuronal survival and dendritic integrity. Additionally, rNPFF significantly upregulated key synaptic proteins, including GAP43, PSD95, and synaptophysin, which are essential for synaptic repair and plasticity. Post-injury rNPFF treatment led to a significant upregulation of pro-brain-derived neurotrophic factor (BDNF) and mature BDNF, which play critical roles in neuronal survival, growth, and synaptic plasticity. Moreover, rNPFF activated the protein kinase Cε isoform, Sirtuin 1, and peroxisome proliferator-activated receptor gamma pathways, which are crucial for regulating cellular stress responses, synaptic plasticity, and energy homeostasis, further promoting neuronal survival and recovery. These findings suggest that rNPFF may play a pivotal role in enhancing neuronal survival and synaptic plasticity after ischemic injury, highlighting its potential as a therapeutic target for stroke recovery.

## 1. Introduction

Cerebral stroke is defined as a sudden interruption of blood supply to the brain, resulting in significant neuronal injury [1,2]. It is one of the primary causes of death and long-term disability globally, severely impacting patients’ survival and quality of life [3]. Stroke often leaves survivors with profound disabilities, including motor deficits and cognitive impairments, which contribute to a substantial socioeconomic burden [3,4]. Despite advancements in acute stroke management, such as thrombolytic therapy and mechanical thrombectomy, many patients do not fully recover, highlighting the limitations of current treatment options [1,4].

Recent research has highlighted the significant impact of epigenetic alterations, especially DNA methylation, on both the pathophysiology and recovery of stroke. DNA methylation occurs when a methyl group attaches to cytosine residues within the DNA, often leading to the suppression of gene expression. This modification is controlled by DNA methyltransferases (DNMTs), such as DNMT1, DNMT3a, and DNMT3b, and can be undone by ten-eleven translocation enzymes [5,6]. Modulating DNA methylation has shown promise in promoting neuroprotection and neurorepair post-stroke. For instance, DNMT inhibitors, such as 5-aza-2′-deoxycytidine (5-Aza-dC), have demonstrated the ability to stimulate neurogenesis, reduce infarct size, and improve functional recovery by preventing aberrant DNA methylation [5,7,8]. This treatment has the potential to regulate the levels of critical synaptic proteins such as synaptophysin and SH3 and multiple ankyrin repeats protein 2, which are essential for synaptic plasticity and cognitive function [8]. Additionally, it can reduce the hypermethylation of genes involved in inflammatory responses and oxidative stress, thereby mitigating neuronal damage and supporting recovery [5].

In addition to epigenetic modulation, task-specific training (TST) has emerged as an effective rehabilitation strategy for enhancing motor recovery and neuroplasticity following stroke. TST involves repetitive practice of a specific task, which induces cortical reorganization and improves motor function related to the practiced task [9,10,11]. Studies have shown that TST can lead to increased dendritic complexity, synaptic plasticity, and functional improvements in both human and animal models of stroke [6,9,11,12]. Numerous research findings indicate that proteins such as BDNF, GAP43, PSD95, and synaptophysin play critical roles in neurogenesis, synaptic repair, and neural plasticity, all of which are crucial for functional recovery post-stroke [6,13,14,15,16].

The combining of DNA methylation inhibition with TST has shown synergistic effects in promoting stroke recovery, enhancing neural plasticity more effectively than either approach alone [6]. In earlier work, we showed that combining TST with the DNMT inhibitor 5-Aza-dC led to significant improvements in functional recovery in a chronic stroke model [6]. This combined treatment was associated with increased mature BDNF levels and enhanced axonal plasticity in the contralesional cortex. This suggests that integrating epigenetic modulation with rehabilitative strategies could optimize stroke recovery outcomes by promoting functional recovery through these mechanisms [6]. Building on these results, identifying new molecules involved in promoting stroke recovery in the contralesional cortex under combined treatment conditions has become increasingly important.

In addition to epigenetic modifiers like 5-Aza-dC, there has been growing interest in peptides with neuroprotective properties, particularly cell-penetrating peptides (CPPs). Several CPPs, such as transactivators of transcription and poly-arginine peptides, have demonstrated neuroprotective effects by facilitating intracellular delivery and promoting neuronal survival under ischemic conditions [17,18]. These peptides have been shown to exert their protective effects by enhancing endocytosis and reducing excitotoxic damage through modulation of ion channels and cellular receptors [19].

Neuropeptide FF (NPFF, FLFQPQRFamide) was originally isolated from bovine brain tissue in 1985 and belongs to the RFamide group of neuropeptides, which are characterized by the common arginine-phenylalanine-amide (Arg-Phe-NH2) motif [20,21]. NPFF and other peptides in this family have been identified in various species, from mammals such as bovine to human cell lines, highlighting their evolutionary conservation and functional significance [20,22,23]. NPFF plays a key role in regulating various physiological functions, such as pain modulation, cardiovascular regulation, neuroendocrine signaling, body temperature regulation, food intake, and blood pressure control [24,25,26]. NPFF exerts its effects through two G protein-coupled receptors, NPFFR1 and NPFFR2. NPFFR1 is primarily found in regions including the hypothalamus and limbic system, whereas NPFFR2 is abundant in the superficial layers of the spinal cord, playing a key role in nociception and opioid modulation [27,28,29,30]. Despite its well-documented role in pain regulation, the potential neuroprotective functions of NPFF, especially in the context of stroke recovery, remain underexplored.

However, recent research has begun to indicate that NPFF may support neural regeneration and promote axonal growth. This research demonstrated that recombinant NPFF protein (rNPFF) promotes corneal nerve injury recovery by rescuing the activation of Sirtuin 1 (SIRT1) and peroxisome proliferator-activated receptor gamma (PPARγ), which are crucial for cellular stress responses and mitochondrial function [24]. Additionally, NPFF may contribute to reducing inflammation, a key factor in stroke pathology [25]. However, further research is needed to fully elucidate the mechanism by which NPFF influences neural recovery. In addition, although a direct link between NPFF and essential neuroplasticity and synaptic repair pathways in functional recovery after stroke has not been studied, NPFF may have the potential to influence these pathways.

We hypothesize that NPFF enhances neuronal survival, promotes synaptic repair, and facilitates synaptic plasticity under ischemic conditions. The aim of this study is to validate the effects of NPFF, identified under stroke recovery-promoting conditions, in photothrombosis-induced rat stroke models treated with 5-Aza-dC combined with TST. To achieve this, we utilized rNPFF in primary mixed cortical cultures subjected to oxygen-glucose deprivation (OGD) and reoxygenation as an in vitro ischemic model, investigating rNPFF’s role in enhancing neuronal survival, synaptic repair, and synaptic plasticity following ischemic injury. This approach aims to establish NPFF as a novel therapeutic target for stroke recovery.

## 2. Results

### 2.1. The Combination of 5-Aza-dC and TST Treatment Enhanced Motor Function Recovery Following a Stroke

The Montoya staircase test is commonly used to evaluate the recovery of motor skills, particularly in reaching and grasping, which are affected by stroke. In this assessment, each animal was presented with 18 food pellets, and the number of pellets successfully retrieved and consumed was documented. Motor function recovery in the affected limb was determined by counting the pellets retrieved by the paw on the side opposite to the stroke lesion [6].

The experimental design included the following groups: a sham control group (C group), a stroke control group (S group), a stroke group that received TST treatment (ST group), a stroke group that was administered 5-Aza-dC (SA group), and a stroke group treated with both 5-Aza-dC and TST (SAT group). A detailed experimental schedule is illustrated in Supplementary Data (File S1).

**Figure 1:**
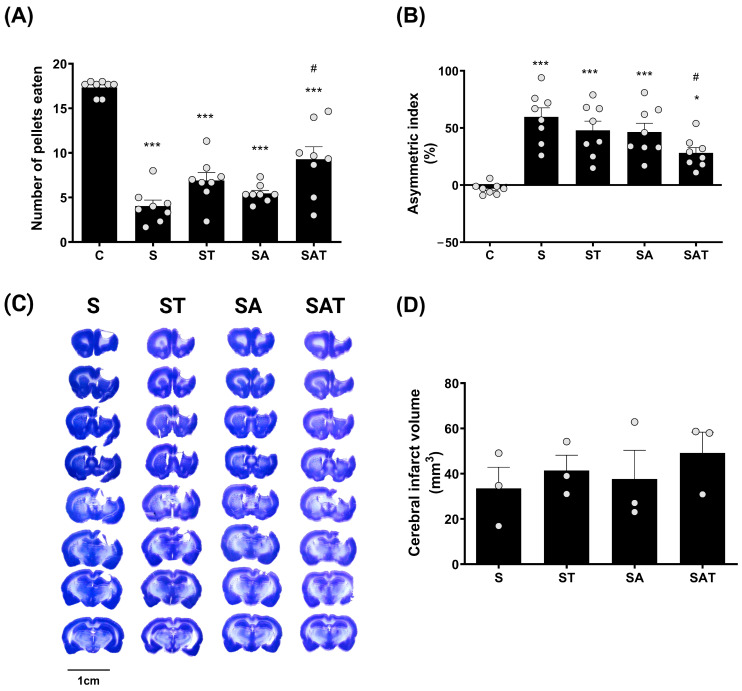
Effect of DNA methylation and TST on motor recovery and infarction size in photothrombotic stroke model. (**A**) The staircase test and (**B**) the cylinder test show motor recovery across experimental groups. Results are presented as the mean ± SEM (n = 8/group). (**C**) Representative Nissl-stained coronal sections showing infarction size across the four experimental groups. Scale bars = 10 mm. (**D**) Quantitative analysis of Nissl staining. Results are presented as the mean ± SEM (n = 3/group). * *p* < 0.05; *** *p* < 0.001 vs. C group. # *p* < 0.05 vs. S group. TST, task-specific training; C, sham control group; S, stroke control group; ST, stroke group treated with TST; SA, stroke group treated with 5-Aza-dC; SAT, stroke group treated with both 5-Aza-dC and TST; SEM, standard error of mean.

One week after treatment, the results of the staircase test revealed a marked decline in the performance of the impaired forelimb in the stroke groups (S, ST, SA, and SAT) when compared to the control group (C). Of these, the SAT group showed the greatest recovery, exhibiting a significant improvement in forelimb function relative to the S group (*p* < 0.05, Figure 1A).

This indicates that the combination of 5-Aza-dC and TST plays a critical role in enhancing motor recovery following stroke.

The cylinder test results showed that, one week after treatment, the usage of the impaired forelimb was significantly reduced in the S, ST, and SA groups (*p* < 0.001) and in the SAT group (*p* < 0.05), compared to the control group. However, this reduction was significantly ameliorated in the SAT group compared to the S group (*p* < 0.05, Figure 1B).

These findings suggest that the combined therapy of DNA methylation inhibition and TST has a positive impact on both skilled and spontaneous movements of the impaired forelimb post-stroke.

### 2.2. Infarct Volume Was Unaffected by the Combination of TST and 5-Aza-dC After Stroke

To assess the impact of the combined therapy on infarct size, we analyzed infarcted brain tissue using Nissl staining. The data revealed that there were no notable differences in infarct size between the groups (Figure 1C,D).

These findings indicate that neither DNA methylation regulation nor TST significantly affect infarct size after ischemic stroke.

### 2.3. Transcriptomic Profiling via RNA Sequencing (RNA-Seq) in Stroke Rats Treated with 5-Aza-dC and TST

#### 2.3.1. RNA-Seq Transcriptome Analysis Findings

**Figure 2:**
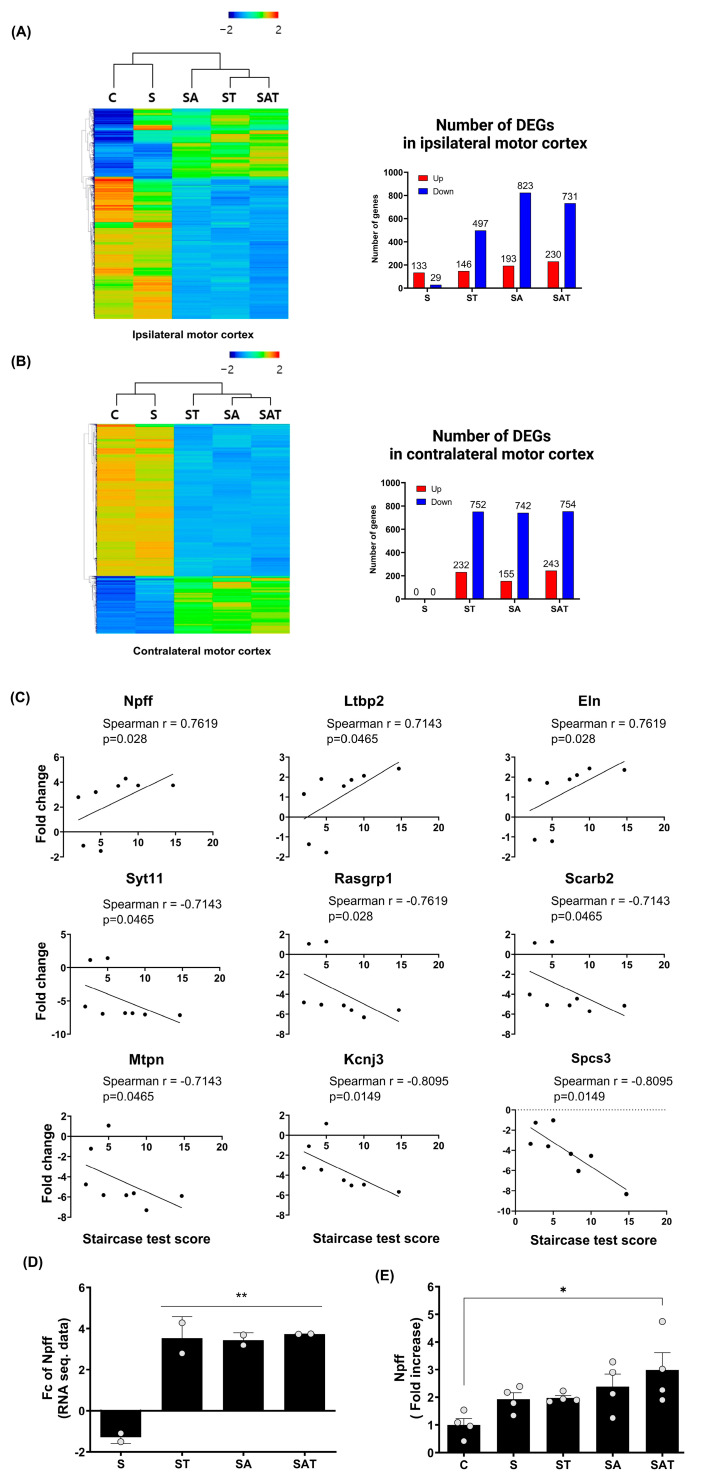
Differentially expressed genes and recovery correlated gene analysis in ipsilateral and contralateral motor cortex. (**A**,**B**) Heat map of hierarchical clustering using z-scores for normalized gene expression values (log2 based) in the ipsilateral (**A**) and contralateral (**B**) motor cortex across the experimental groups (C, S, ST, SA, SAT; n = 2/group). The color scale represents relative gene expression, with red indicating upregulation and blue indicating downregulation. Bar graphs represent the number of differentially expressed genes (DEGs) in the ipsilateral (**A**) and contralateral (**B**) hemispheres for each group compared to the control. (**C**) Spearman correlation analysis between DEG expression in the contralateral motor cortex and behavioral recovery, identifying nine genes with significant correlation. (**D**) Fold change analysis of Npff expression in the contralateral motor cortex across the experimental groups. Results are presented as the mean ± SEM (n = 2/group). ** *p* < 0.01 compared to the S group. (**E**) Quantitative RT-PCR validation of Npff expression, confirming fold changes observed in prior analyses. Results are presented as the mean ± SEM (n = 4/group). * *p* < 0.05 compared to the C group. C, sham control group; S, stroke control group; ST, stroke group treated with task-specific training (TST); SA, stroke group treated with 5-Aza-dC; SAT, stroke group treated with both 5-Aza-dC and TST. DEGs, differentially expressed genes; RT-PCR, reverse transcription polymerase chain reaction; NPFF, neuropeptide FF.

The RNA-seq transcriptome analysis in the motor cortex of stroke rats treated with 5-Aza-dC combined with TST revealed distinct gene expression patterns compared to rats in the control and stroke groups (Figure 2A,B). Hierarchical clustering heat maps visualized the gene expression in the ipsi-lesional and contra-lesional motor cortex after stroke. The number of differentially expressed genes (DEGs) in the ipsi-lesional motor cortex and contra-lesional motor cortex among the study groups is presented in Figure 2A,B, respectively.

#### 2.3.2. NPFF Is Significantly Correlated with Motor Recovery After Stroke

Our comparison of the ipsi-lesional and contra-lesional motor cortex revealed that stroke alone did not induce significant changes in DEG in the contra-lesional cortex. However, unique gene expression patterns emerged in response to drug treatment and TST. To investigate the relationship between these gene expression changes and motor recovery, as measured by the Staircase test, we performed a Spearman correlation analysis focusing on the contra-lesional hemisphere. From this analysis, we identified nine genes with the greatest fold change differences between the SAT and S groups, as the SAT group exhibited the most pronounced recovery compared to the S group (Figure 2C).

In our analysis, we identified nine genes that were significantly correlated with motor recovery following stroke: Npff, latent transforming growth factor beta-binding protein 2, and elastin showed positive correlations, whereas synaptotagmin 11, RAS guanyl releasing protein 1, scavenger receptor class B member 2, myotrophin, potassium inwardly rectifying channel subfamily J member 3, and signal peptidase complex subunit 3 exhibited negative correlations. Since 5-Aza-dC is known to inhibit DNA methylation, potentially leading to increased gene expression, we focused on genes that showed positive correlations with motor recovery. Among these genes, Npff demonstrated a significant correlation with motor recovery (Spearman r = 0.7619, *p* = 0.028) and showed a notable fold change difference between the S and SAT groups (Figure 2D. Fold change difference = 5.03 ± 0.20). Consequently, Npff was identified as a key gene potentially contributing to motor function recovery after stroke. To confirm the findings from the RNA-seq analysis, we carried out quantitative real-time RT-PCR on Npff (Figure 2E). This analysis showed a marked rise in its expression within the SAT group, further corroborating its role in motor recovery (Fold increase = 2.98 ± 0.63, *p* < 0.05 relative to the control group).

### 2.4. rNPFF Treatment Improved Cell Viability After Oxygen-Glucose Deprivation (OGD) and Reoxygenation-Induced Cellular Injury in Primary Mixed Cerebral Cortical Cells

Having identified NPFF as a potential contributor to motor function recovery post-stroke, we proceeded to investigate its neuroprotective effects in vitro. Using primary mixed cortical cultures, we explored the impact of rNPFF treatment on cell survival and synaptic integrity following OGD and subsequent reoxygenation, a widely recognized model for ischemic injury. An overview of the experimental timeline is provided in the Supplementary Materials (File S2).

**Figure 3:**
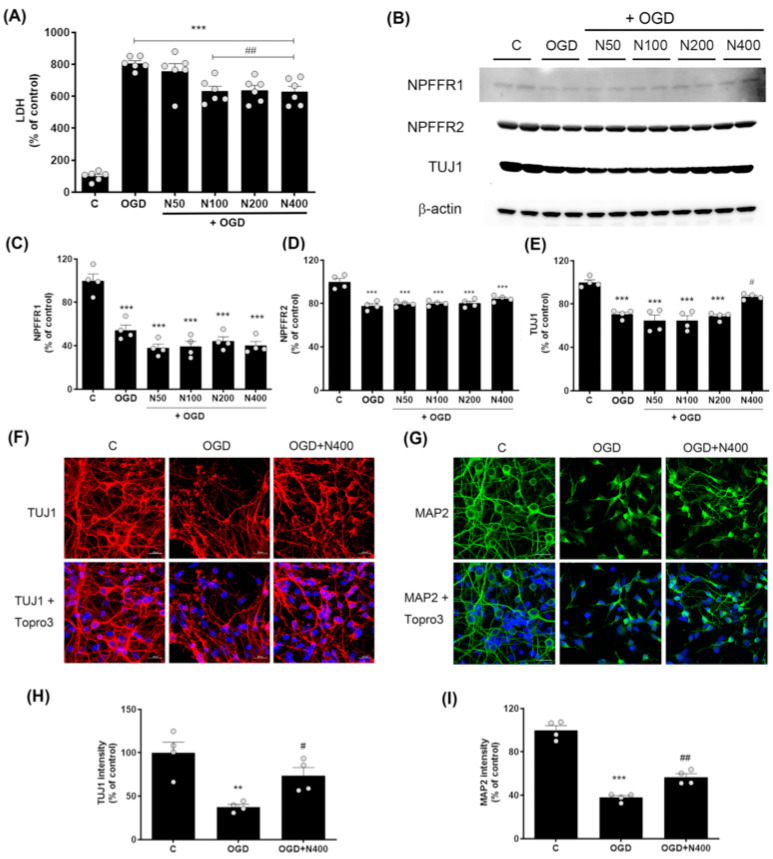
Effects of rNPFF on neuronal injury and recovery in an OGD model. (**A**) Quantification of LDH release in the following groups: control (**C**), OGD, and OGD treated with different concentrations of rNPFF (rNPFF 50 ng/mL, N50; rNPFF 100 ng/mL, N100; rNPFF 200 ng/mL, N200; rNPFF 400 ng/mL, N400). Results are presented as the mean ± SEM (n = 6/group). *** *p* < 0.001 compared to control; ## *p* < 0.01 compared to the OGD group. (**B**) Western blot analysis showing protein expression levels of NPFFR1, NPFFR2, and TUJ1 across groups (C, OGD, and OGD groups with NPFF treatment). (**C**–**E**) Densitometric analysis of NPFFR1 (**C**), NPFFR2 (**D**), and TUJ1 (**E**) expression levels, normalized to control levels. Results are presented as the mean ± SEM (n = 4/group). *** *p* < 0.001 compared to the C group; # *p* < 0.05 compared to the OGD group. (**F**,**G**) Representative immunofluorescence images of TUJ1 (red) (**F**) and MAP2 (green) (**G**), with Topro3 (blue) staining in the C, OGD, and OGD groups treated with N400. Scale bars = 20 μm. (**H**,**I**) Quantification of TUJ1 (**H**) and MAP2 (**I**) fluorescence intensity relative to control levels. Results are presented as the mean ± SEM (n = 4/group). ** *p* < 0.01 compared to the C group; # *p* < 0.05 compared to the OGD group for TUJ1; *** *p* < 0.001 compared to the C group; ## *p* < 0.01 compared to the OGD group for MAP2. C, sham control group; OGD, oxygen–glucose deprivation; rNPFF, recombinant neuropeptide FF protein; LDH, lactate dehydrogenase; SEM, standard error of mean.

To investigate the neuroprotective effect of rNPFF, we measured lactate dehydrogenase (LDH) release, an indicator of cell damage, in cells subjected to 6 h of OGD followed by 18 h of reoxygenation. rNPFF treatment began at the start of reoxygenation and continued for 18 h, with rNPFF administered at concentrations of 50, 100, 200, and 400 ng/mL (Figure 3).

LDH levels were markedly higher in the OGD group (806.87 ± 16.55%, *p* < 0.001) relative to the control group. However, this elevated LDH level was reduced by rNPFF treatment, with significant decreases observed at concentrations of 100 ng/mL (631.86 ± 29.91%, *p* < 0.01), 200 ng/mL (638.14 ± 30.20%, *p* < 0.01), and 400 ng/mL (628.75 ± 33.51%, *p* < 0.01), compared to the OGD group.

This demonstrates that rNPFF shows dose-dependent neuroprotective effects, with significant reductions in cell death observed from 100 ng/mL and higher concentrations compared to the OGD group.

### 2.5. rNPFF Treatment Did Not Alter the Expression of NPFF Receptors 1 and 2 After OGD Plus Reoxygenation

To determine if the neuroprotective effects of rNPFF are mediated through its known receptors, we analyzed the expression levels of NPFF receptors 1 and 2 following OGD and reoxygenation.

The analysis showed that both NPFFR1 and NPFFR2 expression were substantially decreased following OGD (*p* < 0.001). However, rNPFF treatment did not noticeably alter the levels of either receptor (NPFFR1 or NPFFR2). The results revealed no significant changes in the expression of either receptor following rNPFF treatment (Figure 3).

### 2.6. rNPFF Treatment Increased TUJ1 and MAP2 Expression, Enhancing Neuronal Survival and Dendritic Integrity Following OGD Plus Reoxygenation

Given the role of TUJ1 and MAP2 as markers of neuronal survival and dendritic integrity, we investigated whether rNPFF treatment could enhance the expression of these proteins, thereby supporting neuronal integrity under ischemic conditions.

Western blot analysis revealed a marked rise in TUJ1 levels within the group treated with rNPFF at 400 ng/mL compared to the ischemic condition (OGD group) (Figure 3). In the OGD group, TUJ1 expression was recorded at 70.65 ± 1.96% that of the control group, whereas rNPFF treatment at 400 ng/mL increased the expression to 86.84 ± 1.32% of control (*p* < 0.05 compared to the OGD group).

To further confirm the changes in TUJ1 expression observed in the Western blot results, immunocytochemistry analysis was performed. In addition to TUJ1, we also analyzed MAP2, a marker of dendritic structure, to evaluate neuronal integrity.

OGD led to a substantial decrease in the levels of both TUJ1 and MAP2. Specifically, TUJ1 expression was reduced to 37.63 ± 2.90% of control levels (*p* < 0.01), whereas MAP2 expression decreased to 37.89 ± 1.82% of control (*p* < 0.001).

Following rNPFF treatment at 400 ng/mL, the expression levels of both markers increased, with TUJ1 reaching 73.61 ± 9.31% of OGD levels (*p* < 0.05) and MAP2 reaching 56.64 ± 3.15% of OGD levels (*p* < 0.01). These findings indicate that rNPFF enhances both neuronal survival and dendritic integrity under ischemic stress.

### 2.7. rNPFF Treatment Increased Synaptic Marker Expression Following OGD Plus Reoxygenation

Recognizing the critical roles of proteins such as GAP43, PSD95, and synaptophysin in synaptic plasticity and recovery following stroke, we aimed to assess whether rNPFF treatment could increase the expression of these markers, thereby supporting synaptic integrity under ischemic conditions.

**Figure 4:**
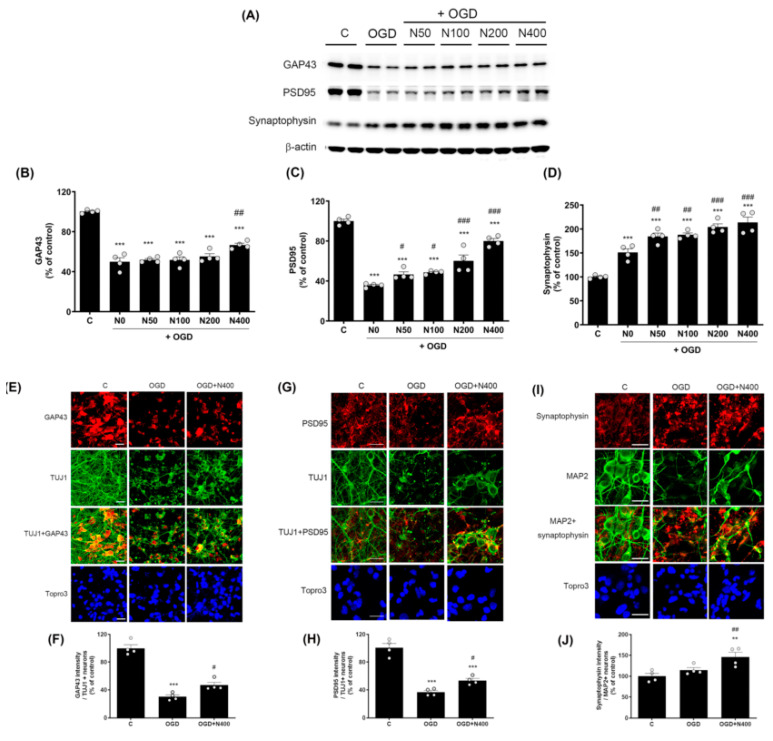
Effects of rNPFF on synaptic plasticity an OGD model. (**A**) Representative Western blots showing the expression of synaptic plasticity markers GAP43, PSD95, and synaptophysin across experimental groups (C, OGD, N50, N100, N200, N400). (**B**–**D**) Densitometric analysis of (**B**) GAP43, (**C**) PSD95, and (**D**) synaptophysin, expressed as a percentage of control levels. Results are presented as the mean ± SEM (n = 4/group). *** *p* < 0.001 compared to the C group; # *p* < 0.05, ## *p* < 0.01, ### *p* < 0.001 compared to the OGD group. (**E**) Representative images of immunofluorescence staining for GAP43 (red) and TUJ1 (green) in the C, OGD, and N400 groups. Merged images and nuclear staining (Topro3, blue) are shown. Scale bars = 20 μm. (**F**) Quantification of GAP43 intensity in TUJ1-positive neurons. Results are presented as the mean ± SEM (n = 4/group). *** *p* < 0.001 compared to the C group; # *p* < 0.05 compared to the OGD group. (**G**) Representative images of PSD95 (red) and TUJ1 (green) staining in the C, OGD, and N400 groups, including merged images and nuclear staining (Topro3, blue). Scale bars = 20 μm. (**H**) Quantification of PSD95 intensity in TUJ1-positive neurons. Results are presented as the mean ± SEM (n = 4/group). *** *p* < 0.001 compared to the C group; # *p* < 0.05 compared to the OGD group. (**I**) Representative images of synaptophysin (red) and MAP2 (green) staining in the C, OGD, and N400 groups. Merged images and nuclear staining (Topro3, blue) are shown. Scale bars = 20 μm. (**J**) Quantification of synaptophysin intensity in MAP2-positive neurons. Results are presented as the mean ± SEM (n = 4/group). ** *p* < 0.001 compared to the C group; ## *p* < 0.01 compared to the OGD group. C, sham control group; OGD, oxygen–glucose deprivation; N50, rNPFF 50 ng/mL; N100, rNPFF 100 ng/mL; N200, rNPFF 200 ng/mL; N400, rNPFF 400 ng/mL; SEM, standard error of mean; rNPFF, recombinant neuropeptide FF protein.

To investigate whether rNPFF influences not only provide neuroprotection but also synaptic plasticity and synaptic recovery, we analyzed the expression of GAP43, PSD95, and synaptophysin through Western blotting (Figure 4).

The expression of GAP43, a protein associated with neural regeneration and axonal growth, was significantly reduced to 49.94 ± 4.04% in the OGD group relative to the C group (*p* < 0.001). However, rNPFF of 400 ng/mL significantly increased GAP43 expression to 66.65 ± 1.87% (*p* < 0.05 when compared to the OGD group).

Similarly, PSD95, a critical protein for synaptic plasticity and structural stability, was reduced to 35.65 ± 0.99% in the OGD group (*p* < 0.001 relative to the C group). rNPFF treatment led to a dose-dependent elevation in PSD95 levels, with notable increases observed at all doses (*p* < 0.05 for rNPFF 50 ng/mL and 100 ng/mL; *p* < 0.001 for rNPFF 200 ng/mL and 400 ng/mL, compared to the OGD group).

Synaptophysin, a marker of synapse formation and neurotransmission, was significantly elevated in all groups relative to the control (*p* < 0.001). Additionally, synaptophysin expression showed a notable rise in all rNPFF-treated groups compared to the OGD group, with a clear dose-dependent effect (*p* < 0.05 for rNPFF 50 ng/mL and 100 ng/mL; *p* < 0.001 for rNPFF 200 ng/mL and 400 ng/mL).

In addition to the Western blot analysis, immunocytochemistry was conducted to further validate the impact of rNPFF on the expression of GAP43, PSD95, and synaptophysin under ischemic conditions. The analysis was conducted using the highest dose of rNPFF (400 ng/mL) to evaluate the changes in synaptic markers under ischemic conditions. To assess changes in synaptic marker expression in neurons, we specifically measured the intensity of the synaptic markers GAP43, PSD95, and synaptophysin in TUJ1+ or MAP2+ neurons.

Immunocytochemistry revealed that rNPFF treatment markedly enhanced the expression of all three synaptic markers. Specifically, GAP43 expression, which was reduced to 30.34 ± 2.59% in the OGD group (*p* < 0.001 relative to the C group), was significantly increased to 46.91 ± 3.98% following rNPFF administration (*p* < 0.05 versus the OGD group). Similarly, PSD95 expression decreased to 36.80 ± 2.49% in the OGD group (*p* < 0.001 relative to the C group) but increased to 53.58 ± 2.99% post rNPFF treatment (*p* < 0.05 versus the OGD group). For synaptophysin, rNPFF administration led to a marked increase, reaching 146.29 ± 11.17%, showing a significant upregulation compared to both the C and OGD groups (*p* < 0.01 for both).

These results suggest that rNPFF treatment enhances synaptic recovery and neuroplasticity in addition to its neuroprotective effects following ischemic stress.

### 2.8. rNPFF Treatment Elevated the Expression of Pro-Brain-Derived Neurotrophic Factor (proBDNF) and Mature Brain-Derived Neurotrophic Factor (BDNF) Following OGD Plus Reoxygenation

**Figure 5:**
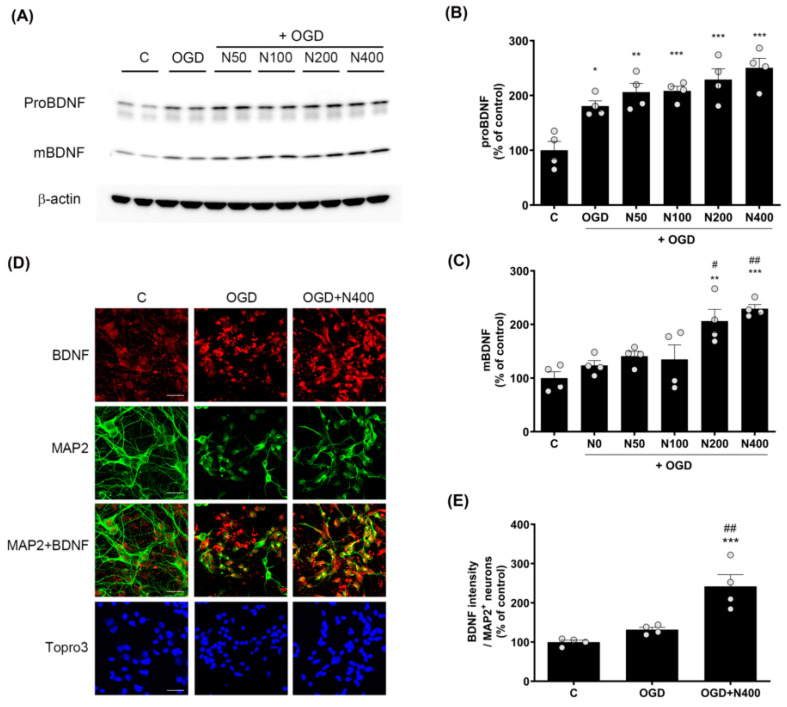
Effects of rNPFF on proBDNF and mBDNF expression in an OGD model. (**A**) Representative Western blots showing the expression of proBDNF and mature BDNF (mBDNF) across the control (**C**), OGD, and rNPFF-treated groups (N50, N100, N200, N400). (**B**,**C**) Densitometric analysis of proBDNF (**B**) and mBDNF (**C**) levels, normalized to control levels. Results are presented as the mean ± SEM (n = 4/group). * *p* < 0.05, ** *p* < 0.01, *** *p* < 0.001 compared to the C group; # *p* < 0.05, ## *p* < 0.01 compared to the OGD group. (**D**) Representative immunofluorescence images showing BDNF (red) and MAP2 (green) staining in the C, OGD, and N400 groups. Merged images of BDNF and MAP2 indicate co-localization in neurons. Nuclear staining with Topro3 (blue) is also shown. Scale bar = 20 μm. (**E**) Quantification of BDNF fluorescence intensity in MAP2-positive neurons. Results are presented as the mean ± SEM (n = 4/group). *** *p* < 0.001 compared to the C group; ## *p* < 0.01 compared to the OGD group. C, sham control group; OGD, oxygen–glucose deprivation; N50: 50 ng/mL; N100: 100 ng/mL; N200: 200 ng/mL; N400: 400 ng/mL; rNPFF, recombinant neuropeptide FF protein; SEM, standard error of mean.

Since BDNF plays a central role in neuroprotection and synaptic plasticity, we evaluated how rNPFF modulates the expression of proBDNF and mature BDNF under ischemic conditions using Western blotting (Figure 5).

The analysis revealed that proBDNF expressions were elevated across all rNPFF-treated groups, including the OGD group, in comparison to the C group. A dose-dependent effect was observed, with statistical significance noted as follows: the OGD group showed a modest increase (180.75 ± 9.65%, *p* < 0.05), while rNPFF 50 ng/mL treatment resulted in a higher expression (206.2 ± 15.93%, *p* < 0.01). The rNPFF 100 ng/mL (208.37 ± 8.67%), 200 ng/mL (229.09 ± 19.76%), and 400 ng/mL (250.38 ± 17.35%) treatments showed the most significant increases (*p* < 0.001 for each).

Additionally, mature BDNF levels were significantly upregulated in the rNPFF 200 ng/mL (206.28 ± 21.81%) and 400 ng/mL (229.26 ± 7.735%) groups compared to both the control and OGD groups. Specifically, mature BDNF levels were significantly increased after rNPFF treatment, with a strong upregulation observed at 200 ng/mL (*p* < 0.01) and 400 ng/mL (*p* < 0.001) compared to the control. Moreover, relative to the OGD group, rNPFF administration led to a marked increase in mature BDNF levels at 200 ng/mL (*p* < 0.05) and 400 ng/mL (*p* < 0.01).

In addition to the Western blot analysis, BDNF expression was also confirmed through immunocytochemistry (Figure 5D and 5E). BDNF expression was measured in MAP2-positive neurons. BDNF expression was elevated in both the OGD group (224.37 ± 11.44%) and the rNPFF 400 ng/mL group (299.39 ± 14.3%) when compared to the control. Importantly, the rNPFF 400 ng/mL displayed a statistically significant increase in BDNF expression in comparison to both the C group (*p* < 0.001) and the OGD group (*p* < 0.01).

This suggests that rNPFF treatment enhances BDNF expression, particularly in MAP2-positive neurons, thereby contributing to its neuroprotective and synaptic plasticity effects. These findings suggest that rNPFF activates BDNF, contributing to neural recovery and functional restoration.

### 2.9. rNPFF Treatment Elevated the Activation of Protein Kinase C (PKC)-Epsilon Isoform (PKCε) Following OGD Plus Reoxygenation

**Figure 6:**
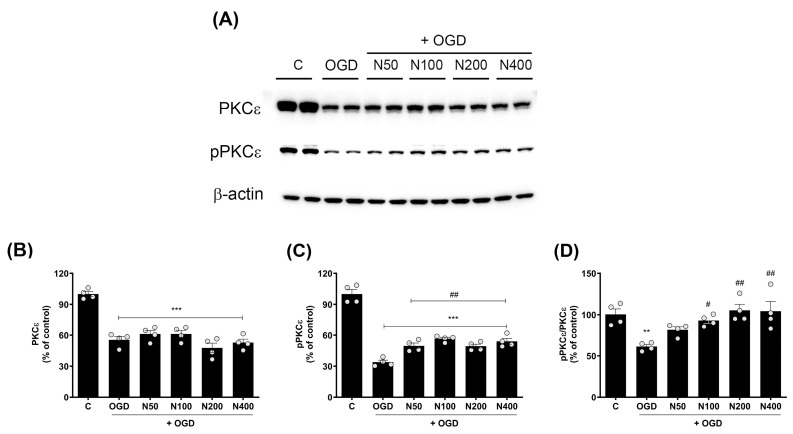
Effects of rNPFF on PKCε and pPKCε expression in an OGD model. (**A**) Representative Western blot images showing the expression of total PKCε and phosphorylated PKCε (pPKCε) across the control (**C**), OGD, and rNPFF-treated groups (N50, N100, N200, N400). (**B**–**D**) Quantification of PKCε and pPKCε expression. Densitometric analysis of PKCε (**B**), pPKCε (**C**), and the pPKCε/PKCε ratio (D), normalized to control levels. Results are presented as the mean ± SEM (n = 4/group). ** *p* < 0.01, *** *p* < 0.001 compared to the C group; # *p* < 0.05, ## *p* < 0.01 compared to the OGD group. C, sham control group; OGD, oxygen–glucose deprivation; N50: 50 ng/mL; N100: 100 ng/mL; N200: 200 ng/mL; N400: 400 ng/mL; rNPFF, recombinant neuropeptide FF protein; SEM, standard error of mean; PKC, protein kinase C.

To explore the broader intracellular signaling mechanisms that may be influenced by rNPFF, we focused on the activation of PKC, a key regulator of synaptic plasticity and neuronal survival. Specifically, we conducted Western blot analyses to assess the expression and phosphorylation status of PKCε (Figure 6), an isoform critically involved in neurotransmitter release and maintaining these synaptic and neuronal functions.

The findings revealed a pronounced decrease in total PKCε expression in the OGD group when compared to the C group (55.54 ± 3.25%, *p* < 0.001), and this reduction remained unchanged even after rNPFF treatment, indicating that NPFF does not influence total PKCε levels in this context.

However, the phosphorylated form of PKCε (pPKCε), which is critical for PKCεs’ activation and function in synaptic signaling and plasticity, was significantly decreased after OGD treatment (33.93 ± 1.95%, *p* < 0.001), but rNPFF treatment administered after OGD significantly restored pPKCε levels in a dose-dependent manner. Specifically, pPKCε levels increased to 49.75 ± 2.66% (*p* < 0.01) with rNPFF 50 ng/mL, 56.67 ± 1.40% (*p* < 0.001) with rNPFF 100 ng/mL, 49.34 ± 2.01% (*p* < 0.01) with rNPFF 200 ng/mL, and 54.11 ± 2.86% (*p* < 0.001) with rNPFF 400 ng/mL.

To quantify the proportion of active PKCε in each group, we analyzed the ratio of phosphorylated PKCε to total PKCε (pPKCε/PKCε), which reflects the activation level of PKCε relative to its total expression. The analysis revealed a significant reduction in the pPKCε/PKCε ratio following OGD treatment (61.29 ± 2.44%, *p* < 0.01), indicating a decrease in PKCε activation. rNPFF treatment after OGD treatment significantly increased this ratio, reaching 93.04 ± 3.33% (*p* < 0.05) with rNPFF 100 ng/mL, 105.3 ± 7.26% (*p* < 0.01) with rNPFF 200 ng/mL, and 104.28 ± 11.59% (*p* < 0.01) with rNPFF 400 ng/mL.

These findings suggest that while rNPFF does not alter total PKCε expression, it enhances the phosphorylation and activation of PKCε after OGD treatment, potentially contributing to synaptic plasticity and neuroprotection under ischemic conditions.

### 2.10. rNPFF Treatment Elevated the Expression of SIRT1 and PPARγ Following OGD Plus Reoxygenation

**Figure 7:**
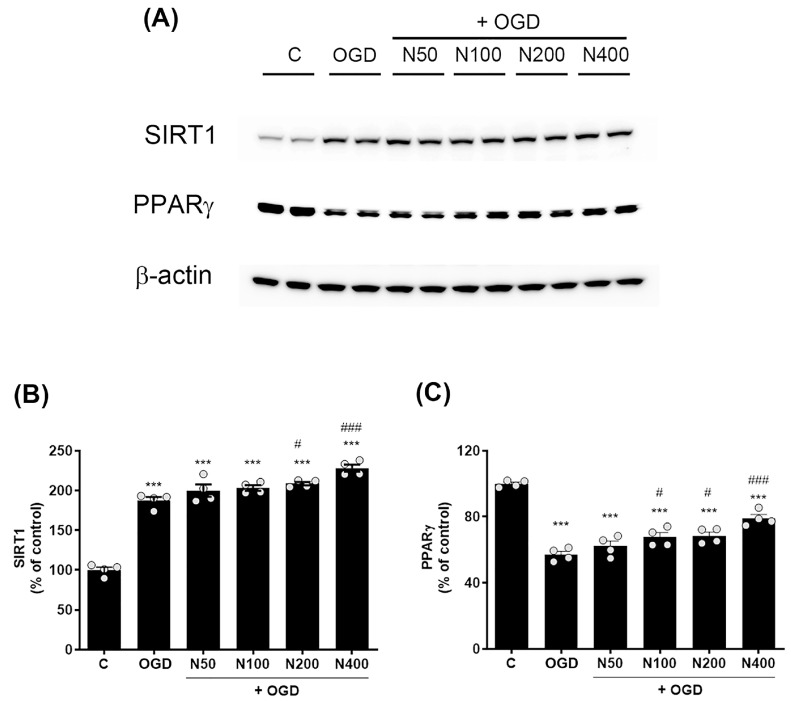
Effects of rNPFF on SIRT1 and PPARγ expression in an OGD model. (**A**) Representative Western blot images showing the expression of SIRT1 and PPARγ across the control (**C**), OGD, and rNPFF-treated groups (N50, N100, N200, N400). (**B**) Quantification of SIRT1 expression. Densitometric analysis of SIRT1 expression, normalized to control levels. Results are presented as the mean ± SEM (n = 4/group). *** *p* < 0.001 compared to the C group; # *p* < 0.05, ### *p* < 0.001 compared to the OGD group. (**C**) Quantification of PPARγ expression. Densitometric analysis of PPARγ expression, normalized to control levels. Results are presented as the mean ± SEM (n = 4/group). *** *p* < 0.001 compared to the C group; # *p* < 0.05, ### *p* < 0.001 compared to the OGD group. C, sham control group; OGD, oxygen–glucose deprivation; N50: 50 ng/mL; N100: 100 ng/mL; N200: 200 ng/mL; N400: 400 ng/mL; rNPFF, recombinant neuropeptide FF protein; SEM, standard error of mean; SIRT1, sirtuin 1; PPARγ, peroxisome proliferator-activated receptor gamma.

To further investigate the mechanisms underlying rNPFF’s neuroprotective effects, we assessed the expression levels of SIRT1 and PPARγ following OGD plus reoxygenation. Western blot analysis showed a pronounced elevation in SIRT1 expression in the OGD group relative to the C group (187.26 ± 4.55%, *p* < 0.001, Figure 7A,B).

rNPFF treatment further elevated SIRT1 levels, with significant increases observed at 200 ng/mL (208.56 ± 2.17%, *p* < 0.05) and 400 ng/mL (228.09 ± 4.45%, *p* < 0.001). In contrast, PPARγ levels were notably reduced in the OGD group relative to the C group (57.12 ± 1.95%, *p* < 0.001, Figure 7A,C). However, rNPFF treatment restored PPARγ levels with notable increases at 100 ng/mL (67.63 ± 2.79%, *p* < 0.05), 200 ng/mL (68.46 ± 2.25%, *p* < 0.05), and 400 ng/mL (79.05 ± 2.32%, *p* < 0.001). In our study, the observed upregulation of SIRT1 and PPARγ suggests that rNPFF may exert its neuroprotective and regenerative effects by activating these key regulatory pathways, which are known to be involved in cellular stress responses, mitochondrial function, and anti-inflammatory processes.

## 3. Discussion

This research explored the neuroprotective properties of rNPFF in the context of stroke, utilizing both transcriptomic analysis and in vitro models of ischemic injury. Initial transcriptomic analysis of stroke-affected animals treated with 5-Aza-dC and TST revealed substantial gene expression changes, with NPFF showing a strong correlation with functional recovery (Figure 1 and Figure 2). This discovery prompted further investigation into rNPFF’s role in promoting neuronal recovery, and we used primary mixed cortical cultures subjected to ischemic injury. Our findings demonstrated that rNPFF not only enhanced cell survival but also upregulated key synaptic proteins and activated signaling pathways essential for neuroprotection and synaptic plasticity. These results highlight the potential of rNPFF as a promising therapeutic candidate for stroke recovery.

One of the most important findings of this study is that rNPFF significantly reduced LDH release, a common marker of cellular damage, in response to OGD and subsequent reoxygenation (Figure 3), indicating a novel role of rNPFF directly mitigating cellular damage in a model of ischemic injury. While RFamide peptides, including NPFF, have traditionally been associated with pain modulation [30] and neuroprotective effects related to opioid-modulating properties in spatial cognition within models of Alzheimer’s disease (AD) [26,31], our study is among the first to reveal rNPFF’s potential neuroprotective role in reducing ischemia-induced neuronal damage and promoting cell survival.

In terms of synaptic integrity, our findings provide novel evidence that rNPFF significantly enhances the expression of key synaptic proteins, including GAP43, PSD95, and synaptophysin, thereby contributing to synaptic repair and plasticity (Figure 4).

The upregulation of GAP43, PSD95, and synaptophysin—proteins known to be critical for synaptic formation and plasticity—suggests that NPFF contributes to synaptic repair. GAP43, for instance, is crucial for axonal growth and neural regeneration [32,33], while PSD95 stabilizes synapses and supports synaptic signaling [16,34]. Synaptophysin, an integral component of synaptic vesicles, is vital for neurotransmitter release, thereby playing a crucial role in maintaining synaptic function during recovery from ischemic injury [35,36,37]. The observed increases in GAP43, PSD95, and synaptophysin expression following rNPFF treatment suggests that rNPFF may promote synaptic repair and plasticity by stabilizing synaptic structures and enhancing signal transduction at both pre- and post-synaptic sites.

Additionally, rNPFF treatment activated critical signaling pathways associated with neuronal survival and synaptic plasticity. In particular, BDNF, which plays an essential role in neuronal survival, growth, and synaptic plasticity, was significantly up-regulated in rNPFF-treated cultures (Figure 5). This study is the first to report that rNPFF treatment leads to an increase in BDNF expression. BDNF is well-known for its role in facilitating neurogenesis and promoting synaptic repair following injury, making its upregulation a key finding in the context of neural recovery [38,39]. BDNF’s involvement in synaptic plasticity is especially relevant in stroke recovery, where synaptic reorganization is critical for motor and cognitive function restoration [38,39,40,41]. This suggests that rNPFF may contribute to the recovery process by enhancing BDNF-mediated pathways, further promoting synaptic stability and functional recovery after ischemic injury.

The activation of PKCε by rNPFF further supports the hypothesis that rNPFF enhances synaptic plasticity through multiple pathways. PKCε is a critical mediator in long-term potentiation, a process essential for learning and memory, and it also plays a pivotal role in modulating neurotransmitter release [42,43]. Our study found that rNPFF restored the levels of pPKCε, a marker of PKCε activation, which were significantly reduced following ischemic injury. This restoration suggests that rNPFF may strengthen synaptic signaling and maintain synaptic integrity under ischemic stress by activating PKCε (Figure 6).

Moreover, the role of PKC in synaptic plasticity extends to its regulation of key synaptic proteins. For instance, GAP43, a protein crucial for axonal growth and neural regeneration, is directly regulated by PKC-dependent phosphorylation. Phosphorylated GAP43 binds to actin filaments with higher affinity, stabilizing them and promoting growth cone extension and motility. This enhanced actin stabilization facilitated by GAP43 phosphorylation is essential for synaptic repair and plasticity [44,45]. Additionally, PKC activation has been linked to the upregulation of other synaptic proteins, such as PSD95 and synaptophysin, which are crucial for maintaining synaptic structure and function [46]. PSD95 stabilizes synapses and supports synaptic signaling, while synaptophysin, an integral component of synaptic vesicles, plays a vital role in neurotransmitter release during recovery from ischemic injury [47].

BDNF, which is significantly upregulated by rNPFF treatment in our study, has been shown to promote the expression of synaptophysin and other synaptic proteins through pathways that may involve PKC activation. This is supported by previous studies indicating that BDNF enhances synaptophysin expression, thus contributing to synaptic plasticity and recovery post-stroke [40]. Our findings are in alignment, as the observed increase in BDNF likely contributes to the upregulation of synaptophysin, reinforcing the synaptic repair mechanisms activated by rNPFF in the context of ischemic injury.

Furthermore, our data revealed that rNPFF activates the SIRT1 and PPARγ pathways, both of which are involved in regulating cellular stress responses and energy homeostasis. SIRT1 is widely recognized for its role in extending cellular lifespan by enhancing DNA repair mechanisms and promoting resistance to oxidative stress [48,49]. The upregulation of SIRT1 observed in our study (Figure 7) suggests that rNPFF’s neuroprotective effects may be partially mediated through this pathway, helping cells to resist ischemic damage and promote recovery through enhanced mitochondrial function.

Similarly, PPARγ, known for its anti-inflammatory and neuroprotective properties, was upregulated by rNPFF in our ischemic model (Figure 7). PPARγ plays a critical role in regulating gene expression linked to mitochondrial biogenesis and inflammatory responses, both of which are key to reducing neuronal damage post-stroke [50,51]. By activating both SIRT1 and PPARγ, rNPFF may not only protect neurons from acute ischemic injury but also enhance long-term survival and function by modulating metabolic and anti-inflammatory pathways. These effects are critical in the context of stroke recovery, where inflammation and metabolic dysregulation can exacerbate neuronal death and impair recovery.

In line with our findings, the diabetic corneal nerve injury model demonstrated that rNPFF not only promotes nerve regeneration but also upregulates SIRT1 and PPARγ [24]. In that study, NPFF levels were significantly lower in diabetic trigeminal sensory neurons, with hyperglycemia contributing to the deficiency of ocular properties in diabetic mice. The administration of rNPFF facilitated the extension of neurites in diabetic trigeminal neurons and, when delivered via subconjunctival injection, supported the healing of corneal nerve injuries. Furthermore, rNPFF treatment restored the activation of SIRT1 and PPARγ in diabetic trigeminal neurons, supporting the idea that rNPFF’s neuroprotective effects extend beyond cellular survival to actively enhance nerve regeneration in both peripheral and central nervous systems.

## 4. Materials and Methods

### 4.1. Animals

Eight-week-old male Wistar rats, weighing an average of 280.45 ± 2.25 g, were obtained from Orient Bio Incorporation (Seongnam, Republic of Korea). The rats were maintained in a room with controlled temperature (23 ± 0.5 °C) under a 12 h light/dark cycle. Food and water were provided ad libitum. Animal experimental procedures were approved by the Animal Experiment Review Board of the Institutional Animal Care and Use Committee (IACUC) of Konkuk University (Permit Number: KU18134). All experiments, including treatment, anesthesia, and euthanasia, were conducted in accordance with ARRIVE guidelines. The authors adhered to the Stroke Therapy Academic Industry Roundtable (STAIR) guidelines for conducting preclinical stroke studies [9].

### 4.2. Photothrombotic Stroke Model

Motor cortical infarcts were induced using the photothrombosis technique [6,9,10,52]. Rats were anesthetized with an intraperitoneal (i.p.) injection of ketamine (50 mg/kg) and xylazine (5 mg/kg) and then positioned in a stereotaxic frame (Stoelting Co., Wood Dale, IL, USA). After exposing the skull, a KL1500 LC cold light source (Carl Zeiss, Jena, Germany) with a 4 mm fiber optic bundle was placed 4.0 mm lateral to the bregma, targeting the right motor cortex. Rose Bengal (Sigma-Aldrich, St. Louis, MO, USA) was administered via i.p. injection at a dose of 10 mg/kg for 5 min prior to activating the light. The light was then switched on for 20 min. Sham animals underwent the same procedure but without the rose bengal injection. Following recovery from anesthesia, the rats were returned to their cages. Following recovery from anesthesia, the animals were regularly monitored for signs of distress, illness, or abnormalities, though no animals were excluded for these reasons in this study. Brain tissue collection occurred 13 days post-stroke, focusing on the infarct core and penumbra, as well as equivalent regions from the contralateral side in injured animals and both hemispheres of control rats.

### 4.3. Animal Grouping

A total of 40 rats were used in this study. Initially, all animals underwent a period of environmental adaptation, followed by a one-week handling process by two experimenters to acclimate them to the experimental conditions. Subsequently, the animals participated in one week of staircase training. Rats that successfully retrieved all 18 pellets during the staircase training period were included in the study. Those that failed to retrieve all 18 pellets within the training period were excluded. However, no rats were excluded, as all successfully completed the task within the allotted time.

After the adaptation and training periods, the animals were divided into two main groups. Eight rats were randomly assigned to the sham control group (n = 8), while the remaining 32 rats underwent photothrombosis surgery to induce ischemic stroke.

On the fourth day post-surgery, motor deficits were evaluated using a staircase test. None of the stroke-induced rats retrieved more than 2 out of 18 pellets, confirming the presence of motor deficits in all 32 rats. Therefore, all stroke-induced rats were included in the study.

On the fifth day post-surgery, the stroke-induced rats were further randomized into four subgroups:

(1) Stroke group (S): This group included 8 rats that did not receive any additional treatment post-stroke induction
(2) Stroke with TST group (ST): This group included 8 rats that received TST for 1 week following the induction of stroke
(3) Stroke with 5-Aza-dC injection group (SA): This group included 8 rats that received 5-Aza-dC injections for five days post-stroke induction
(4) Stroke with combined 5-Aza-dC injection and TST group (SAT): This group included 8 rats that received both 5-Aza-dC injections for five days and TST for 1 week post-stroke induction

For the treatment regimen, starting from the fifth day post-stroke induction, rats received i.p. injections of 5-Aza-dC (0.4 mg/kg) for five consecutive days and underwent TST for seven consecutive days.

### 4.4. Behavior Test

All behavioral experiments were conducted three times. Before photothrombosis surgery, before the initiation of treatment, and after the completion of the treatment.

#### 4.4.1. Staircase Test

For the staircase test, the rats underwent a one-week training period with food restriction while having unrestricted access to water. Their body weight was monitored daily to ensure it did not fall below 85% of their initial weight. Each training session lasted for 15 min, with two sessions conducted per day. During training, the rats were placed in a transparent modified staircase apparatus where they retrieved pellets (Bioserve Inc., Frenchtown, NJ, USA), placed on the steps. The apparatus had six steps on one side, with three pellets on each step, totaling 18 pellets.

Food was withheld from the rats the day before the test. On the test day, the rats were placed in a clear acrylic container and required to use their affected paw to retrieve 18 pellets positioned on the affected side, consuming 3 pellets per step. They were removed from the container and returned to their cages either after consuming all 18 pellets or after 15 min had passed. A total of three trials were conducted over two days. The number of pellets eaten with the injured paw was recorded, and the rats were videotaped throughout the test. The average number of pellets consumed across the three trials was used as the final score [6,53].

#### 4.4.2. Cylinder Test

The cylinder test was employed to measure the spontaneous use of the affected paw [6]. Rats were placed in a transparent cylinder with a diameter of 20 cm, and the number of times the rats touched the walls with their paws over a 5 min period was recorded. The rats’ movements were captured using an overhead camera during the test. The testing area was sterilized with 70% alcohol before each new rat was tested. Functional analysis involved measuring the time (in seconds) each paw—impaired, unimpaired, or both—touched the wall in slow motion (at 1/4th real-time speed). Analysis was only conducted when it was clearly determined that the paws touching the wall were bearing weight. The degree of recovery of the injured paw was expressed as an asymmetry index by calculating the percentage of time each limb touched the wall. The formula used was: Asymmetry index = (% of ipsilateral limb use) − (% of contralateral limb use) [54].

#### 4.4.3. Video Recording

All behavioral tests were recorded using a Sony HDR-CX350 Handycam (Sony, Tokyo, Japan). The Staircase test was filmed from the side to capture the movements of the rat’s paws and mouth, while the Cylinder test was recorded from above to observe the rat’s interaction with the cylinder. After recording, the videos were analyzed using GOM Player v2.3 (Gom & Company, Seoul, Republic of Korea) to assess the rats’ behaviors.

### 4.5. Task-Specific Training (TST)

TST was conducted once daily for one week, starting on day 5 post-stroke induction [6]. The rehabilitation chamber was a transparent acrylic box with a pellet box positioned on the left side [55]. Each rat was placed in the rehabilitation chamber and allowed to consume 300 pellets using its impaired forelimb for a duration of 40 min. Rats were removed from the rehabilitation chamber and returned to their cage either after consuming all 300 pellets or when the 40 min period had elapsed. During the training period, the rats were subjected to a restricted diet but were allowed to drink water freely. The groups that did not undergo TST were fed an amount of regular food equivalent to the weight of 300 pellets. Researchers measured the rats’ body weight daily, ensuring that it did not decrease below 85% of their initial weight.

### 4.6. Regulation of DNA Methylation via 5-Aza-dC Injection

A stock solution of the DNMT inhibitor 5-Aza-dC (A3656, Sigma Aldrich, St. Louis, MO, USA) was prepared by dissolving 10 mg of 5-Aza-dC in 200 µL of DMSO, followed by the addition of 800 µL of saline. The stock solution was aliquoted and stored at −80 °C. For treatment, the stock solution was diluted in saline to a final concentration of 0.4 mg/kg and administered via i.p. injection. The drug was freshly prepared and protected from light during the experimental session. Injections were initiated on day 5 post-stroke induction and continued daily for 5 days. A previous study reported that they achieved the greatest behavioral result at 5 h after 5-Aza-dC i.p. injection [56]. Therefore, rats in the TST combined therapy group received the injection at least 5 h prior to training.

### 4.7. Measurement of Infarction Volume

Following anesthesia with i.p. injections of ketamine (100 mg/kg) and xylazine (10 mg/kg) to ensure deep anesthesia for sacrifice, the animals were perfused transcardially with 0.9% normal saline containing 0.5% sodium nitrite and 10 U/mL heparin sulfate, followed by 4% paraformaldehyde in 0.1M phosphate buffer (pH 7.4), according to previously described methods [52]. Brains were extracted, postfixed in 4% paraformaldehyde for 24 h, and transferred to 30% sucrose in PBS at 4 °C for 48 h. Coronal sections of 40 μm were then prepared at −20 °C using a cryostat and stored at −80 °C in antifreeze solution. Every 20th section, covering the region from bregma −5.2 mm to 2.2 mm, was collected on slides and stained with 0.5% cresyl violet. The intact areas of both the ipsi- and contra-lesional hemispheres were measured using ImageJ (v1.5k, https://imagej.net accessed on 24 July 2021). The volume of the intact hemisphere was calculated by multiplying the intact area by 0.04 (slice thickness in mm) and by 20 (the sectioning interval). Total infarct volume was determined by subtracting the volume of the intact area in the ipsi-lesional hemisphere from that in the contra-lesional hemisphere. Infarct volumes were measured independently by two blinded experimenters [52].

### 4.8. RNA Isolation and Analysis of RNA-Sequencing

For all groups, the peri-infarction area of the ipsi-lesional hemisphere was dissected. Corresponding areas were also collected from the contra-lesional hemisphere. After homogenizing the collected tissues, total RNA was extracted using Trizol reagent (Invitrogen, Grand Island, NY, USA). As described by Choi et al. [52], total RNA was used to construct cDNA libraries following the protocol of the TruSeq Stranded mRNA Sample Prep Kit (Illumina, San Diego, CA, USA). Initially, 1000 ng of total RNA was processed by selecting polyadenylated RNA (mostly mRNA) with oligo-dT magnetic beads. The isolated mRNA was fragmented, and single-stranded cDNA was synthesized using random hexamer primers, with Actinomycin D added to prevent second-strand DNA synthesis. The second strand of cDNA was synthesized by removing the RNA template and incorporating dUTP (deoxyribouridine triphosphate) instead of dTTP (deoxythymidine triphosphate). A single adenine base was then added to the 3′ end of the cDNA to facilitate the ligation of sequencing adapters. These adapters were amplified by polymerase chain reaction (PCR), where amplification only proceeded using the first strand to preserve strand specificity. The quality of the final cDNA libraries was assessed for size distribution using the Agilent Bioanalyzer (DNA 1000 kit; Agilent, Santa Clara, CA, USA) and quantified via qPCR (Kapa Library Quant Kit; Kapa Biosystems, Wilmington, MA, USA), followed by normalization to 2 nmol/L for sequencing [57]. The libraries were sequenced on the HiSeq4000 platform (Illumina, San Diego, CA, USA) by Macrogen Incorporated.

### 4.9. Differential Expression Genes Analysis

We preprocessed the raw sequencing reads to remove low-quality bases and adapter sequences. The processed reads were mapped to the Rattus norvegicus genome assembly (rn6) using HISAT v2.0.515 for alignmen [58]. The reference genome and annotation data were obtained from the UCSC Table Browser (http://genome.ucsc.edu, accessed on 14 February 2022), while transcript assembly and quantification were performed using StringTie v1.3.3b [59,60]. Gene expression levels were calculated in fragments per kilobase of exon per million mapped reads (FPKM). Genes with non-zero FPKM values were retained, followed by log2 transformation and quantile normalization. We employed independent *t*-tests to determine differentially expressed genes (DEGs) and utilized the Benjamini–Hochberg method to control the false discovery rate (FDR). Genes were considered DEGs if their fold change was ≥2 and *p* < 0.05. Spearman correlation analysis was performed to evaluate the association between DEGs in the contralesional motor cortex and motor function recovery (as measured by the staircase test). Genes with high correlation were selected as potential targets.

### 4.10. Quantitative Real-Time PCR

We conducted quantitative real-time PCR (qPCR) to validate RNA-seq results by analyzing the expression of NPFF [61]. Total RNA was isolated from cortical tissues using Trizol reagent (Invitrogen, Carlsbad, CA, USA). Reverse transcription was carried out for 1 h at 42 °C with 50 ng of total RNA, employing 20 units/µL of avian myeloblastosis virus (AMV) reverse transcriptase (Roche Applied Science, Indianapolis, IN, USA) and oligo-p(dT)15 primers. The reaction was stopped by heating at 99 °C for 5 min. PCR amplification was performed using cDNA obtained from 50 ng of total RNA on a LightCycler 480 Instrument II (Roche, NY, USA). The qPCR reaction was set up using the LightCycler 480 SYBR Green I Master kit (Roche, NY, USA), and the reaction targeted rat Npff (NM 022586.1) using the following primers: Forward: AGCTGGGGTGAAGACCAAGT, Reverse: CTGGAGCAGAACACGCATGA. GAPDH (Forward primers: CATGACCACAGTCCATGCCA and reverse primers: ACCAGTGGATGCAGGGATGA) served as the reference gene, and the qPCR data were processed following the manufacturer’s instructions.

### 4.11. Rat Primary Mixed Cerebral Cortical Cell Culture

Following previous methods [62], the cerebral cortices of Sprague–Dawley rat embryos were harvested at embryonic day 18. The tissues were incubated with 0.01% trypsin in Hank’s balanced salt solution for 15 min at 37 °C, followed by gentle pipetting using a Pasteur pipette to dissociate the tissues. After trituration, primary cortical neurons were plated onto polystyrene cover slides pre-coated with 50 μg/mL poly-D-lysine (Sigma Aldrhich, Saint Louis, MO, USA) in culture plates. For immunocytochemistry, cells were seeded in 4-well chambers (Nunc^®^ Lab-Tek^®^ II Chamber Slide, Thermo Fisher Scientific, Waltham, MA, USA) at a density of 3.8 × 10^5^ cells per well with an area of 1.7 cm^2^, using a chamber dimension of 74 × 25 mm. For Western blotting (WB), cells were plated in 60 mm dishes (Thermo Fisher Scientific, Waltham, MA, USA) at a density of 4.8 × 10^6^ cells per dish, with a surface area of 21.5 cm^2^. The cells were cultured at 37 °C in a humidified atmosphere containing 5% CO_2_ using MEM, 1M glucose, 2.5% FBS, 2.5% horse serum, and glutamax-100.

### 4.12. OGD and Reoxygenation Exposure

On the 7th day of in vitro culture, the medium was replaced with glucose-free, serum-free MEM, and the cells were placed in an anaerobic incubator (Forma Anaerobic Systems, Thermo Electron, Norristown, PA, USA) under an environment containing 90% nitrogen, 5% carbon dioxide, 5% hydrogen, and 98% humidity at 37 °C for 6 h. After OGD exposure, the cultures were removed from the anaerobic chamber, the medium was refreshed, and the cells were returned to standard culture conditions. Control cultures were maintained in glucose-supplemented MEM under normal conditions for the same time period [62]. After OGD exposure, cells were reoxygenated for 18 h, and rNPFF treatment was administered simultaneously with the start of reoxygenation.

### 4.13. rNPFF Treatment

Recombinant human pro-FMRFamide-related NPFF partial protein (MyBiosource, San Diego, CA, USA, Catalog Number: MBS1019306, Lot Number: YD04546K1g5) was administered at concentrations of 50, 100, 200, and 400 ng/mL upon reoxygenation initiation. Individual experiments were repeated at least four times using cells obtained from embryos of different pregnant rats.

### 4.14. rNPFF Source and Characterization

According to the certificate of analysis provided by the manufacturer, rNPFF is an N-terminal GST-tagged recombinant protein containing a partial sequence corresponding to human NPFF (66-76aa), with the sequence SQAFLFQPQRF. The protein was expressed in *E. coli* and purified using affinity chromatography. It was prepared in liquid form in a buffer containing 0.2 μm sterile-filtered PBS (pH 7.4) with 50% glycerol. After aseptic processing and endotoxin removal, the protein exhibited endotoxin levels below 1.0 EU per μg, as determined by the limulus amebocyte lysate method. Verified by SDS-PAGE analysis, the protein demonstrated 95% purity and was provided at a concentration of 0.5 mg/mL. This rNPFF was stored at −20 °C, with repeated freezing and thawing avoided. For cell treatment, the liquid rNPFF was directly diluted into the culture media to final concentrations of 50, 100, 200, and 400 ng/mL. The rNPFF remained stable and homogeneous at these concentrations, ensuring consistency across experiments.

### 4.15. Lactate Dehydrogenase Assay

Cell viability was evaluated by detecting lactate dehydrogenase (LDH) activity released into the culture medium using a cytotoxicity assay kit (Promega Bioscience, San Luis Obispo, CA, USA) [62]. In brief, 50 μL of the culture medium was combined with an equal volume of LDH substrate solution (Promega, Madison, WI, USA) and incubated for 30 min. The reaction was then halted by adding 1 M acetic acid (half the reaction volume), and the absorbance at 490 nm was measured using a Spectra Max ABS (Molecular Devices, Menlo Park, CA, USA). Cells cultured under normal conditions served as baseline controls for the LDH activity assay. LDH activity was calculated and presented as a percentage relative to the control group.

### 4.16. Western Blot Analysis

The cells were rinsed with ice-cold PBS and then lysed on ice using RIPA buffer (10% Phosphatase inhibitor, 1% Protease inhibitor cocktail) containing a protease inhibitor mixture (AEBSF, aprotinin, bestatin hydrochloride, E-64, leupeptin, pepstatin A) (Sigma Aldrhich, Saint Louis, MO, USA), and then separated using a homogenizer. A total of 20 µg of soluble protein per lane was loaded onto an SDS-PAGE gel and electrotransferred to a PVDF membrane (Millipore, Burlington, MA, USA) [62]. Specific protein bands were detected using primary antibodies, including NPFFR1 (ABIN6390353; Antibodies, Cambridge, UK), NPFFR2 (ABIN6390354; Antibodies, Cambridge, UK), TUJ1 (801201; Biolegend, San Diego, CA, USA), GAP43 (AB5312; Millipore, Burlington, MA, USA), PSD95 (AB9708; Millipore, Burlington, MA, USA), synaptophysin (611880; BD Biosciences, San Jose, CA, USA), BDNF (ab108319; abcam, Cambridge, UK), PKCε (2683; Cell Signaling Technology, Danvers, MA, USA), Phospho-PKCε (NBP3-13312; Novus, Centennial, CO, USA), SIRT1 (ab110304; abcam, Cambridge, UK), PPAR gamma (PA3-821A; Thermo Fisher Scientific, Waltham, MA, USA); β-actin (A5441; Sigma Aldrich, St. Louis, MO, USA). Following incubation with primary antibodies, the membranes were treated with goat anti-rabbit and goat anti-mouse secondary antibodies (Jackson, Bar Harbor, ME, USA) conjugated to horseradish peroxidase (HRP). Detection was performed using enhanced chemiluminescence (Pierce, Rockford, IL, USA).

We selected specific proteins for this analysis based on their established roles in neuroprotection, synaptic plasticity, and ischemic recovery processes. TUJ1 and MAP2 were assessed as markers of neuronal survival and dendritic integrity [63,64,65]. GAP43, PSD95, and synaptophysin were chosen due to their critical roles in synaptic plasticity and recovery following ischemic injury [32,34,36]. PKCε and its phosphorylated form were measured because PKC signaling is a key regulator of synaptic plasticity and neuronal survival, particularly through its involvement in neurotransmitter release and synaptic maintenance [46,47]. SIRT1 and PPARγ were evaluated for their involvement in cellular stress responses, mitochondrial function, and anti-inflammatory processes, which are important for NPFF’s neuroprotective mechanisms [24,48,49,50,51].

### 4.17. Fluorescence Immunostaining of Cells

Cells were grown in a 4-well chamber slide (Nunc^®^ Lab-Tek^®^ II Chamber Slide, Thermo Fisher Scientific, Waltham, MA, USA) at a density of 3.8 × 10⁵ cells per well, covering an area of 1.7 cm^2^ (dimensions: 74 × 25 mm). They were incubated for 1 h at 24 °C in PBS with 5% horse serum and 0.03% Triton-X100. The slides were then incubated overnight at 4 °C with primary antibodies in PBS containing 2.5% horse serum and 0.01% Triton-X100 [62]. Primary antibodies used in this study included TUJ1 (801201, Biolegend, San Diego, CA, USA), MAP2 (M3696; Sigma Aldrhich, Saint Louis, MO, USA), PSD95 (51-6900; Thermo Fisher Scientific, Waltham, MA, USA), synaptophysin (611880; BD Biosciences, San Jose, CA, USA), GAP43 (AB5312; Millipore, Burlington, MA, USA), and BDNF (ab108319; abcam, Cambridge, UK).

Specific binding was detected by incubating the samples at room temperature for 60 min with Alexa Fluor-conjugated donkey anti-rabbit secondary antibodies (488) diluted 1:200 (Invitrogen, Grand Island, NY, USA). The cells were then washed with 0.1 M PBS and stained with Topro3 (Invitrogen, Grand Island, NY, USA). The mounted slides were analyzed using a confocal microscope (LSM 710, Carl Zeiss, Oberkochen, Germany) with fluorescence emissions captured at 546 and 647 nm. A minimum of four randomly captured images were analyzed per group. Neuronal signal intensity analysis was performed using Image J (v1.5k, https://imagej.net, accessed on 22 November 2023), with measurements taken from four randomly selected regions of interest (ROIs) of 159.73 µm × 159.73 µm each. The total area analyzed corresponded to approximately 0.025 mm^2^ within a 4-well chamber with a surface area of 1.7 cm^2^.

### 4.18. Data Analysis and Statistics

The staircase test and cylinder test were analyzed using one-way analysis of variance, followed by Tukey’s multiple comparison test for post hoc analysis. A one-way ANOVA was also used to compare the infarct volume, intensity of Western blot results, intensity of immunofluorescence staining, LDH measurements, and RT-qPCR results, with Tukey’s multiple comparison test applied as the post hoc test in each case. Spearman correlation analysis was conducted to determine the correlation between DEG expression in the contralesional hemisphere and the results of the staircase test. All values are presented as the mean ± standard error. A *p*-value of less than 0.05 was considered statistically significant for rejecting the null hypothesis. Data analyses were conducted using GraphPad Prism version 10.3.1. (GraphPad Software, Boston, MA, USA). Hierarchical clustering and heatmaps were generated using MultiExperiment Viewer (MeV) (https://webmev.tm4.org/about, accessed on 14 February 2022) version.

## 5. Conclusions

Collectively, our findings suggest that rNPFF exerts a dual neuroprotective effect by reducing cell death and promoting synaptic recovery. This is consistent with the broader role of neuropeptides in providing protection against neuronal damage and enhancing plasticity. Additionally, by activating key molecules and regulating cellular activity through pathways involving BDNF, PKCε, SIRT1, and PPARγ, rNPFF orchestrates a comprehensive neuroprotective response that supports both neuronal survival and synaptic plasticity. Therefore, our study highlights rNPFF as a promising therapeutic target for stroke recovery.

Future research should explore rNPFF’s potential for long-term recovery in vivo and investigate its interactions with other neuroregenerative processes, including its role in neuroinflammation and oxidative stress reduction. These factors are vital components of both acute and chronic stroke recovery, where modulating immune responses and oxidative stress is crucial for improving outcomes. Our study investigated the effects of rNPFF in a mixed cortical culture model simulating ischemic conditions, with a primary focus on its role in neurons. However, further research is needed to explore how rNPFF influences other cell types under these conditions, particularly glial cells. Moreover, additional studies are necessary to identify other signaling pathways beyond the mechanisms we have identified that may also contribute to rNPFF’s neuroprotective effects. Furthermore, as future studies continue to explore NPFF’s potential in promoting motor recovery, this research could serve as a foundation for clinical therapeutic applications.

## Data Availability

All relevant data are included within the article.

## Supplementary Materials

The following supporting information can be downloaded at: https://www.mdpi.com/article/10.3390/ijms252111580/s1.
