# Supplementary figures and images for: Neuropeptide FF Promotes Neuronal Survival and Enhances Synaptic Protein Expression Following Ischemic Injury
Source: Int J Mol Sci. 2024 Oct 28;25(21):11580. doi: 10.3390/ijms252111580 (PMC11546865; doi:10.3390/ijms252111580)

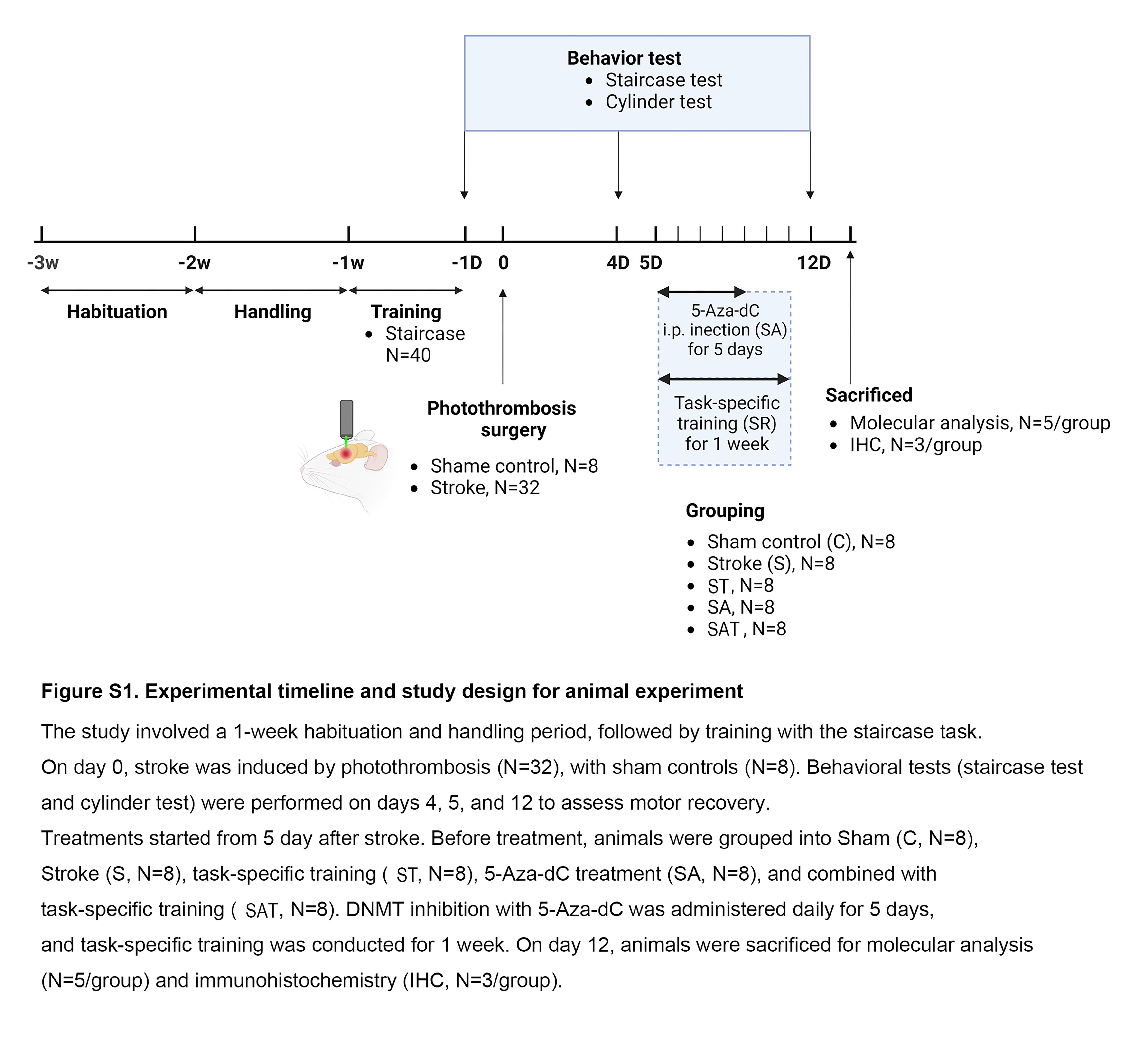

Supplement: Supplementary file 1 [file ijms-25-11580-s001.zip › Figures S1& revised S2 600dpi/FigureS1.tif]
